# Brain Radiation Information Data Exchange (BRIDE): integration of experimental data from low-dose ionising radiation research for pathway discovery

**DOI:** 10.1186/s12859-016-1068-8

**Published:** 2016-05-11

**Authors:** Christos Karapiperis, Stefan J. Kempf, Roel Quintens, Omid Azimzadeh, Victoria Linares Vidal, Simonetta Pazzaglia, Dimitry Bazyka, Pier G. Mastroberardino, Zacharias G. Scouras, Soile Tapio, Mohammed Abderrafi Benotmane, Christos A. Ouzounis

**Affiliations:** Department of Genetics, Development & Molecular Biology, School of Biology, Aristotle University of Thessalonica, 54124 Thessalonica, Greece; Institute of Radiation Biology, Helmholtz Zentrum München, German Research Center for Environmental Health GmbH, 85764 Neuherberg, Germany; Radiobiology Unit, Belgian Nuclear Research Centre (SCK•CEN), B-2400 Mol, Belgium; School of Medicine, IISPV, “Rovira i Virgili” University, Sant Llorens 21, 43201 Reus, Spain; Laboratory of Radiation Biology & Biomedicine, Agenzia Nazionale per le Nuove Tecnologie, l’Energia e lo Sviluppo Economico Sostenibile (ENEA) Centro Ricerche Casaccia, 00123 Rome, Italy; National Research Center for Radiation Medicine of the National Academy of Medical Sciences of Ukraine, Melnykov str. 53, Kyiv, 04050 Ukraine; Erasmus Medical Center, 3015GE Rotterdam, The Netherlands; Biological Process & Computation Laboratory (BCPL), Chemical Process & Energy Resources Institute (CPERI), Centre for Research & Technology Hellas (CERTH), Thessalonica, 57001 Greece; Present address: Department of Biochemistry and Molecular Biology, University of Southern Denmark, Campusvej 55, 5230 Odense M, Denmark

**Keywords:** Low-dose ionising radiation, Data integration, Brain research, Omics technologies, Systems biology

## Abstract

**Background:**

The underlying molecular processes representing stress responses to low-dose ionising radiation (LDIR) in mammals are just beginning to be understood. In particular, LDIR effects on the brain and their possible association with neurodegenerative disease are currently being explored using omics technologies.

**Results:**

We describe a light-weight approach for the storage, analysis and distribution of relevant LDIR omics datasets. The data integration platform, called BRIDE, contains information from the literature as well as experimental information from transcriptomics and proteomics studies. It deploys a hybrid, distributed solution using both local storage and cloud technology.

**Conclusions:**

BRIDE can act as a knowledge broker for LDIR researchers, to facilitate molecular research on the systems biology of LDIR response in mammals. Its flexible design can capture a range of experimental information for genomics, epigenomics, transcriptomics, and proteomics. The data collection is available at: <bride.azurewebsites.net>.

## Background

In recent years, industrial societies have experienced a significant increase of exposure to low-dose ionising radiation (LDIR), with possible implications for human health and disease [1]. The known causes of LDIR exposure typically arise from advanced medical diagnostic procedures [2], air travel [3] and nuclear industry incidents, including the major Chernobyl [4] and Fukushima [5] disasters. Other effects might involve specific population groups, for instance health professionals with frequent exposure to ionising radiation or space travelers [6]. Examples of acute doses that motivate much of LDIR research include medical diagnostic procedures or radiotherapy treatment. It is estimated that, in total, the annual increase to LDIR exposure has dramatically risen on average from 0.5 mSv in 1980 to 3 mSv today, particularly in the industrial world [7]. This general trend stipulates the intensification of research on LDIR effects on health – both chronic and acute [8], in particular the understanding of molecular mechanisms involved with a view to radiation protection as well as the mitigation of those effects by policies or precautionary measures at low- or even moderate-ionising radiation doses [9].

Since the early days of LDIR research, questions regarding health effects at the molecular and system levels have been raised [10–12]. Early studies with variable doses concentrated on certain tissues, e.g. skin [13] or bone [14] and molecules, e.g. thioredoxins [15]. Subsequently, comparisons between normal and neoplastic cell lines [16] and studies of cellular processes such as apoptosis [17] have contributed towards a deeper appreciation of the complex responses to LDIR, with implications for human health [18] or specific situations, e.g. air [3] or space [19] travel.

Despite significant progress, it was not until a decade ago that a better understanding has emerged with regard to the underlying molecular processes involved in the LDIR response [20]. The most pertinent studies have highlighted those effects with low dose for skin [21] and higher doses for the cardiovascular system [22, 23] – first recorded in tissue culture and later as models for human physiology at the whole-tissue level. The genome-wide quest for reliable biomarker molecules for radiation exposure has been instigated recently [24, 25], with focus on individual molecules [26, 27], proteomics at high [28, 29] or low [30, 31] doses, and expression studies [32–34] or particular conditions, for example effects on neurodegenerative disease [35, 36]. To our knowledge, the lowest doses ever published for radiation effects involve 20 mGy for mouse heart [37] and liver [38].

While LDIR effects for skin or heart have been extensively recognized, very little is currently known for their action in the cerebrovascular system and the brain [9, 39]. To access this black box of human physiology, integrated approaches with mouse models and molecular, cellular, organismal, behavioral, and epidemiological components are becoming vital [40]. These complex data landscapes need to be organized and analyzed using proper data integration platforms – by merging relevant databases, experimental resources, analytical tools and systems biology [41].

## Construction and content

### Data integration requirements

In our efforts to record and analyze relevant experimental and computational information for LDIR effects on the brain, we have taken a light-weight approach to data integration [42]. Previously, several approaches have attempted to address critical bottlenecks in the integration of complex biological data, such as disregard of commonly accepted data standards, variable user interfaces, lack of collaborative spaces, immature data exchange services and time consuming pipelines for advanced bioinformatics analysis [43]. The continuing increase of data volumes creates additional obstacles in both processing and analysis. The concept of big data combined with cloud services [44] provides a direction for new solutions to the above mentioned challenges.

There are several ways of achieving integration between data resources, including biological databases and lab data collections. First, the data warehouse concept proposes the creation of a local data repository to facilitate queries executed locally; second, the single-database engine approach offers more efficient access via queries, which however need to be executed locally using full indexing; third, hypertext link integration provides opportunities for less structured collections, with the predictable drawback of complex data navigation [45]. The rise of web technologies contributes towards the development of new protocols and platforms called Web Services that maintain a middle ground between the above options, in order to exchange data between different data resources or systems. Typical examples of such approaches are based on Service-Oriented Architectures (SOAs) [46] or REpresentational State Transfer (REST) [47].

In our work, the integration of data resources in the context of LDIR research presented two challenges: first, to assemble relevant publicly available omics data – including transcriptomics and proteomics for a number of conditions, tissues and phenotypes under consideration, and second, to include novel experimental data from collaborating laboratories within a framework that will lead to molecular systems biology-based pathway inference and biomarker discovery. Thus, the high-level requirements for data integration in LDIR research in non-technical language are: the recording of the identity of relevant molecules (i.e. with sequence identifiers), the quality control of the imported data from the literature and own experiments, and the secure transfer and access of those data by partner laboratories and researchers, as well as a public access portal.

In line with the primary aims and the four high-level requirements mentioned above, we have developed a platform for LDIR research called BRIDE. BRIDE provides access to a number of tasks displayed as tabs for users, including editing gene lists and tools, while implementing light-weight integration with a number of hand-picked, relevant web services for computational systems biology, including genomics, transcriptomics, proteomics and phenotype data resources [41].

We have thus combined results recorded from an exhaustive analysis of the existing literature with our own experimental results. We have created ‘unification links’ connecting molecules with their corresponding database entries and ‘relationship links’ connecting molecules with their biological context [48], associating them with co-expressed genes, protein interactions or cellular pathways [49]. BRIDE supports access via a web browser client [50]. This type of integration is called navigational or link-based, and can be ideal for development efforts with modest resources [45].

## System architecture

The BRIDE platform implementation is based on the Microsoft® (MS®) computational ecosystem. According to our extensive research at the early development phase, there are several examples with successful implementations of bioinformatics projects using MS® solutions [51]. Thus, we have decided to implement a scalable, robust, industry-strength solution for BRIDE. The development was attained with Visual Studio 2012 [52], using in particular the LightSwitch tool. From the architectural point of view, the BRIDE platform is a browser client, 3-tier system [53]. This deployment scenario creates an application that runs via the end-user’s web browser. The database and server components run on a database server, running on web MS® IIS server.

The LightSwitch component executes widely-used MS® technologies and patterns like Entity Framework for data access, n-tier application layers and Model-View-View-Model (MVVM) [54]. This type of architecture allows for any system component to be modified without having to change the other two parts of the 3-tier architecture, therefore facilitating maintenance and response to changes. The tiers of the platform communicate through interfaces. Therefore, as long as the interface remains stable, the internals can change without affecting the rest of the platform.

**Fig. 1:**
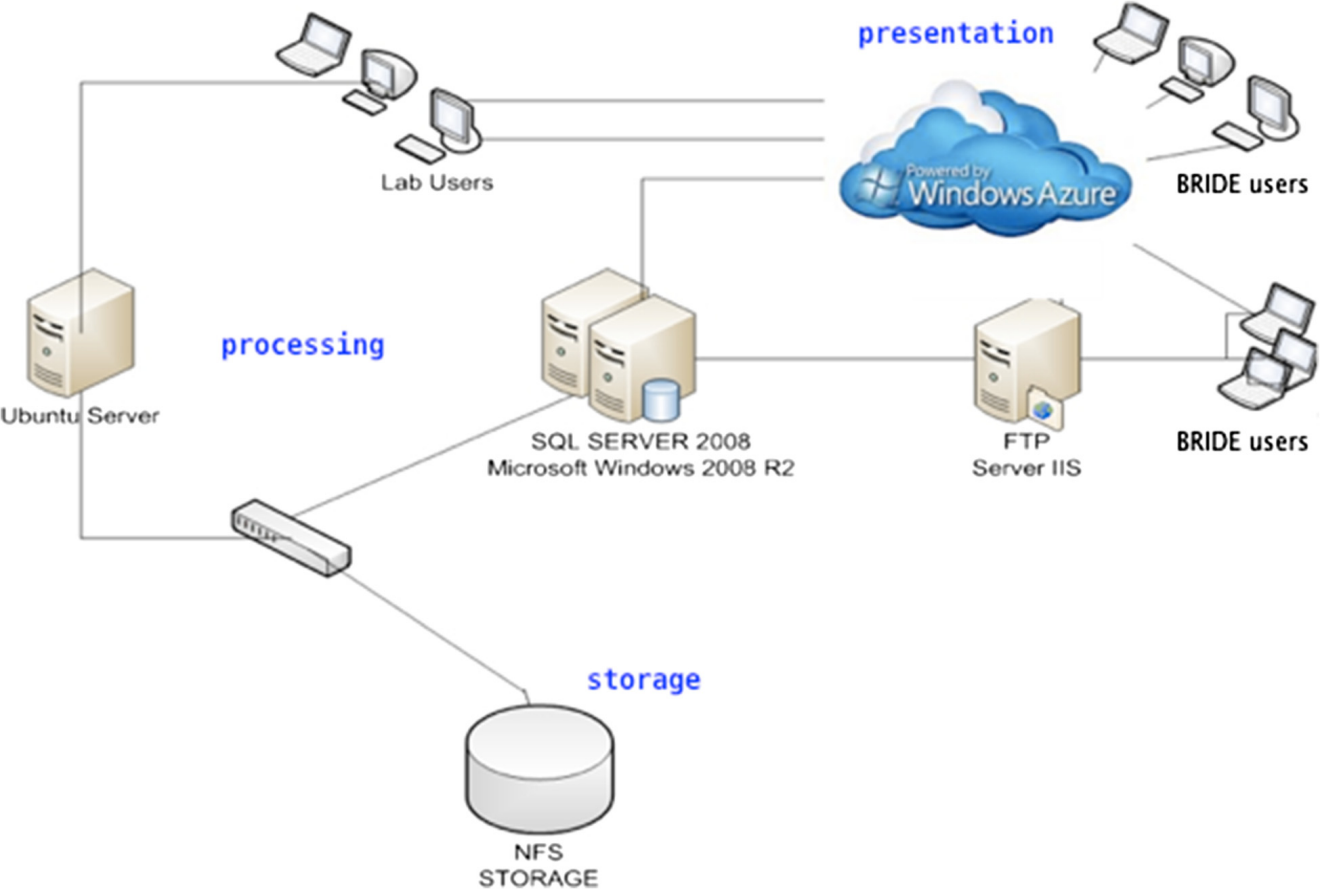
BRIDE system architecture. The 3-tier architecture between storage, processing and presentation

With 3-tier applications, the business rules^1^ and queries are removed from the client and are executed on a system between the user interface (e.g. client browser) and the data storage system (in this case, the MS® SQL database). The client application provides a standard user interface or a presentation layer for the platform. The business rules server ensures that all of the business processing and queries is executed properly, and serves as an intermediary between the client and the data storage layers. Note that, in this type of application, the client does not access the data storage system directly. The final deployment is based on a MS® platform: local servers run the Windows® 2008 R2 Server operating system, FTP server, MS® SQL 2012 Server relational database management system and MS® Azure cloud services and can be replicated at other sites (Fig. 1). According to our usage pattern, planning the hybrid cloud solution fits optimally to our needs: this solution ensures data security, with the database hosted locally, while the presentation layer or the web site is hosted on the cloud. The main benefits for the cloud-based services are lower costs, a significant decrease in development time and low system administration workloads.

The steps in the data preparation process were as follows: (i) partner laboratories submitted their experimental results to the BRIDE storage site via a secure FTP server – accepted formats are MS® Excel files or tab-delimited text files. The recommended fields were: gene name (used from the corresponding reference genome), organism (mouse in this case), tissue (brain or other tissues), time after irradiation, radiation dose (metadata) and lab (identification); (ii) using Google Refine [55], an open source power tool for cleaning large data sets, we were able to convey and store data, using http requests and stripping techniques, and (iii) subsequently enrich the submitted collections with identification names from different resources – e.g. UniProt protein identifiers [56] based on gene names; (iv) the final table is exported to MS® SQL production database; (v) finally, we have generated all ‘unification’ links using SQL store procedures. The data preparation stage offered a seamless pipeline to prepare the data for submission. Using SQL store procedures, it is easy to update links in case a data provider issues any alterations to their web-services definitions.

### Data consumption

**Fig. 2:**
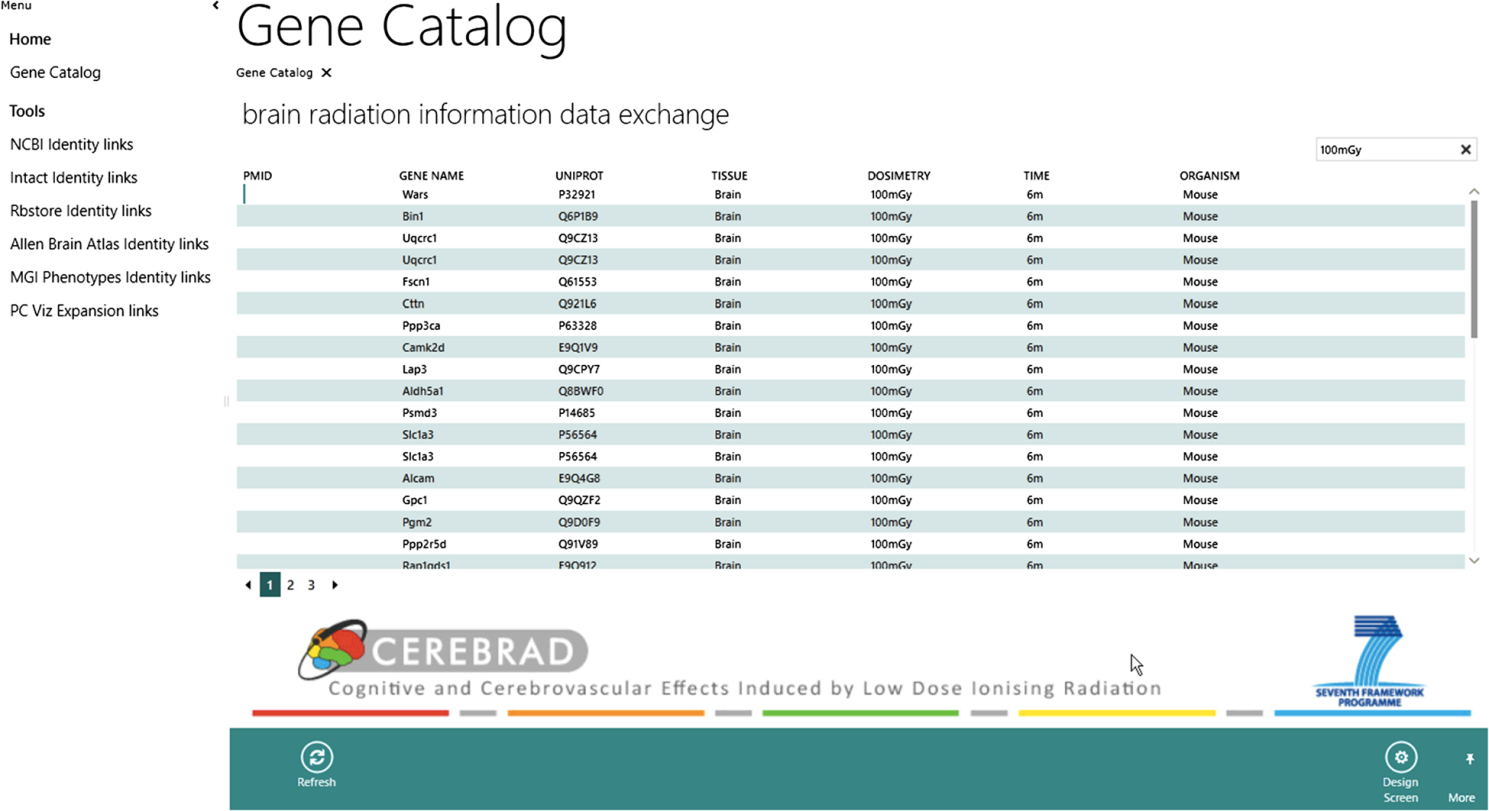
BRIDE gene catalog. A detailed screenshot of BRIDE’s gene catalog, with entities described

End users are thus able to access all BRIDE data at two different levels: (i) Users can access a fully searchable gene catalog via their browser, through unification or relationship links. ‘Unification links’ connect gene entries with their corresponding database records [48]. We have also managed to integrate data contents from NCBI resources [57], the IntAct protein interaction database [58], the Allen Brain Atlas [59] and Rb-STORE (www.rbstore.eu/) into BRIDE, with a view to continue capturing information from molecular resources against a rich backdrop of phenotypic features relevant to systems radiobiology, as needs arise (Fig. 2). The links within BRIDE, and across data resources, were built using the available REST APIs, which are distinct for each data repository. The corresponding actions (input/output) for unification links are listed in Table 1. ‘Relationship links’ connect molecules with their local biological context, as mentioned above [48]. Users may select more than one molecule and search for their respective pathways using the PCViz component of the PathwayCommons resource [60], accessible at <pathwaycommons.org/pcviz/>.

**Table 1:** BRIDE integration with data resources

Provider	Section	Type	Results
NCBI	Graph	Unification Link	web page
	Fasta	Unification Link	web page
	Biosystems	Unification Link	web page
	Pathway	Unification Link	web page
	Protein	Unification Link	web page
	Geo Profile	Unification Link	web page
	PIE	Unification Link	web page
EMBL-EBI IntAct	Cytoscape Graph	Unification Link	cytoscape file
	Ch EBI Ontology Browser	Unification Link	web page
	GO Ontology Browser	Unification Link	web page
	Taxonomy Browser	Unification Link	web page
	Interactions	Unification Link	web page
Rb Store	Organism	Unification Link	web page
	Tissue	Unification Link	web page
Allen Mouse Brain	Mouse Brain Experiments	Unification Link	web page
	Developing Mouse Brain Experiments	Unification Link	web page
	Expression Mask Image	Unification Link	image file
PCViz	Pathway Commons Network Visualizer	Relationship Link	web page

(ii) The second method available for BRIDE data consumption is based on the MS® Open Data (OData) technical protocol [61]. OData defines an abstract data model allowing different clients to access those data programmatically. OData builds on AtomPub, an abstract implementation of a REST design pattern, ignoring some of the REST constraints in the process. OData services require URIs construction to enable the protocol querying capability and returns results in XML (Fig. 3) or JSON formats [62]. The benefit of this protocol implementation is that users can access data in a high-throughput mode or use tools like the MS® Excel plugin Pivot, in order to import data and proceed to further analysis locally – e.g. by including those data into the popular Ingenuity® suite.

**Fig. 3:**
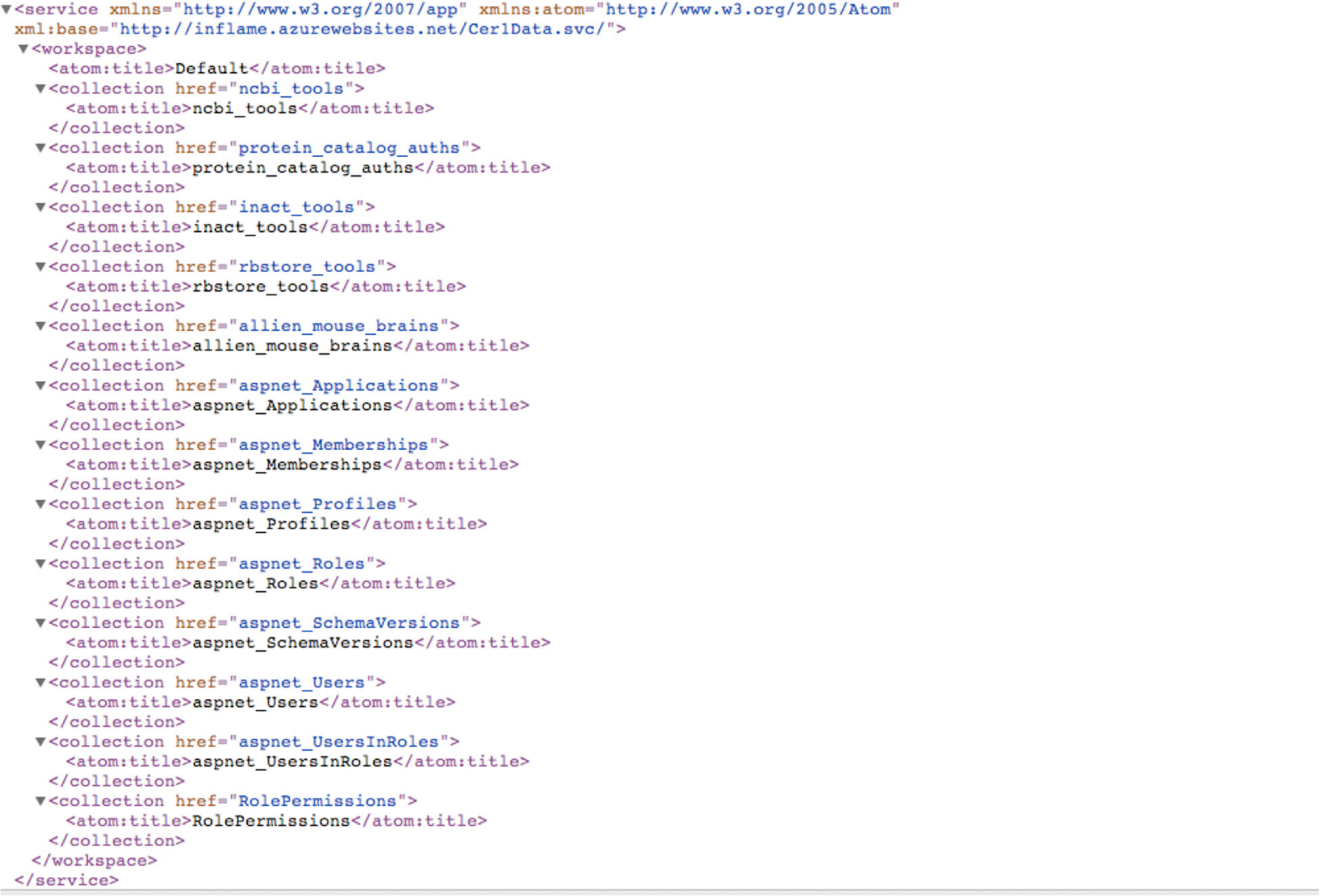
OData XML results. Example results in XML format

To achieve the above goals following the specified user requirements, in particular to provide a systems biology context for the LDIR response at the molecular level, one needs to first identify the molecules under consideration and second to understand their interactions with other molecules at different levels of expression. Coupled with the experimental efforts within the CEREBRAD project for transcriptomics and proteomics, we plan to explore this landscape of relevant molecules and interactions to better understand LDIR response in the brain (manuscript in preparation).

### Data contents

Currently, the BRIDE collection contains 3,174 relevant records defined uniquely by the tuple: ‘tissue-dose-time (after irradiation)’. The majority of these entries (3,016, or 95 %) correspond to protein-coding genes detected in the mouse brain, according to the original studies (recorded as PubMed identifiers – PMIDs) – this does not necessarily mean that they are brain-specific. A small minority of proteins are found in other tissues as well, and have been included due to their mentions in the same experimental recordings. These entries might further be used as controls in brain studies – for instance, inclusion of those entries in future brain-related studies. Most records are compiled from four publications associated with the CEREBRAD project, referred again by their PMID numbers, 3009 in total (or 95 %): more precisely, 533 [35], 182 [63], 1828 [64], 312 [65] and another 154 unpublished instances. The remaining 165 instances have been recorded manually by scanning over hundreds of relevant articles in the literature, and selecting six additional publicatoins – their PMIDs are also provided [28, 29, 49, 66–69]. For all these gene entries, unification and relationship links were generated, where possible. Averaging ~18 links per molecule (Table 1), we have >55,000 links at our disposal. Thus, the corpus of data within BRIDE is extremely rich as well as challenging to explore, for pathway inference in the context of radiation effects on the brain, e.g. cognitive deficits in adult, prenatally exposed mice [70, 71]. These links also provide critical histology and other experimental evidence for the involvement of the corresponding genes in brain function, e.g. the Allen Mouse Brain Atlas.

The other two elements defining uniqueness of the recorded entries in the BRIDE data collection, namely dosimetry and time, are less uniform thus reflecting a wide range of experimental designs or conditions (Table 2): for instance, 48 % of records refer to doses <1 Gy while 46 % of records have been observed at more than 5 weeks since irradiation. A small number (133 in total) of entries were not assigned to a specific dosimetry-time as these observations came from the scanner literature, with unclear experimental details (but are recorded for completess and can be filtered out).

**Table 2:**
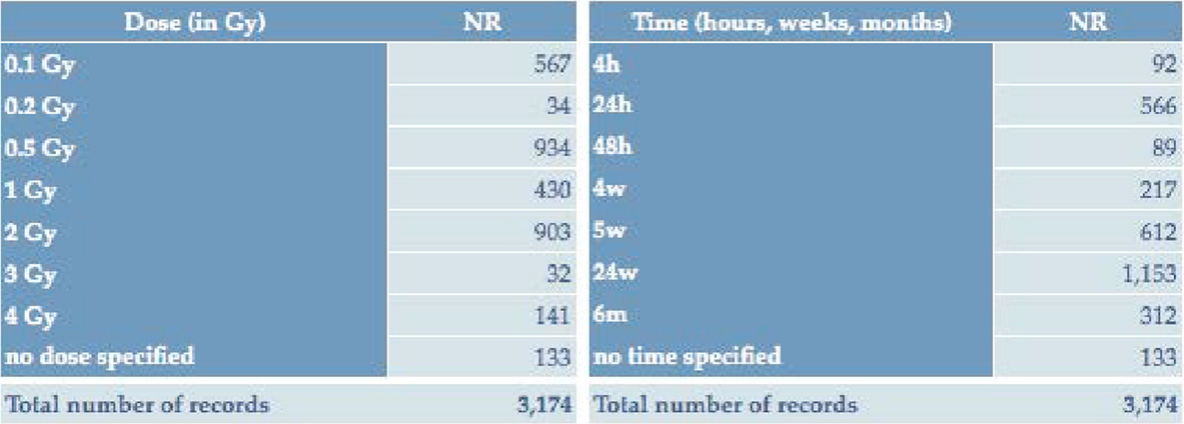
Dose and Time statistics for all records in the BRIDE data collection

## Utility and discussion

The BRIDE platform is an easy-to-use resource with a clean design, modest development efforts, and wider applicability in radiobiology research that supports our joint efforts and distributes the obtained results to the wider community. We have primarily taken a gene/protein-oriented approach with the view that a genome browser-like design would be both labor-intensive and of unclear relevance at this exploratory phase of LDIR response. Database development and implementation have been based on modern software technologies and protocols. The fundamental design principles were platform usability and portal access, which expand data consumption options available to end users. We have thus minimized the effort of platform management by utilizing a hybrid cloud deployment method. The automated data preparation pipeline currently allows scientists to focus on their studies and not wrangle with data formats. Finally, Uniform resource identifier (URI) integration links are easily updated in case of web service modifications by data providers. Further integration with other parallel efforts, such as Radiation Genes [72] or NIF [73], might be possible in the future.

## Conclusion

The BRIDE platform can act as a knowledge broker for LDIR researchers, to cope with the ever-increasing amounts of data, their heterogeneous nature, the varying landscape of data types and formats, and the expanding resources for genomics, epigenomics, transcriptomics, and proteomics in LDIR research – as well as a community data portal. As the project evolves, we will understand better the requirements and improve the peer-to-peer communication of scientific results between stakeholders, with the aspiration that BRIDE is used widely by the LDIR community and beyond [74]. While we are still in the process of analyzing these results and other omics aspects of LDIR response (manuscript in preparation), the BRIDE data integration platform design already allows direct use and design modifications that can capture additional types of information from next-generation sequencing (NGS), as well as epigenomics and behavioral data.

## Availability and requirements

The BRIDE platform can be accessed with a web browser at <bride.azurewebsites.net>.

## Consent

No ethics approval was required for this work, as all results reported have already been published elsewhere.

## Abbreviations

mGy: 1/1000 Gray
mSv: 1/1000 Sievert

## Footnotes

1 This is a technical term, does not correspond to the colloquial use of the word.
